# Comparison of *in vivo* knee kinematics before and after bicruciate-stabilized total knee arthroplasty during squatting

**DOI:** 10.1186/s12891-021-04669-9

**Published:** 2021-09-12

**Authors:** Masato Kiyohara, Satoshi Hamai, Hirotaka Gondo, Hidehiko Higaki, Satoru Ikebe, Ken Okazaki, Yasuharu Nakashima

**Affiliations:** 1 Department of Orthopaedic Surgery, Graduate School of Medical Sciences, Kyushu University, 3-1-1 Maidashi, Higashi-ku, Fukuoka, 812-8582 Japan; 2 Department of Medical-Engineering Collaboration for Healthy Longevity, Kyushu University, 3-1-1 Maidashi, Higashi-ku, Fukuoka, 812-8582 Japan; 3 Department of Life Science, Faculty of Life Science, Kyushu Sangyo University, 2-3-1 Matsugadai, Higashi-ku, Fukuoka, 813-8503 Japan; 4 Department of Creative Engineering, National Institute of Technology, Kitakyushu College, 5- 20-1 Shii, Kokuraminami-ku, Kitakyushu, Fukuoka 802-0985 Japan; 5 Department of Orthopaedic Surgery, Tokyo Women’s Medical University, 8-1 Kawada-cho, Shinjyuku-ku, 162-8666 Tokyo, Japan

**Keywords:** Total Knee Arthroplasty, Bicruciate-stabilized, Preoperative Kinematics, Postoperative Kinematics, Image-matching Technique

## Abstract

**Background:**

No studies have directly evaluated kinematic changes during squatting before and after bicruciate-stabilized total knee arthroplasty (BCS-TKA) with the dual cam-post mechanism and asymmetric surfaces. This study investigated the effect of BCS-TKA on changes to pre- and postoperative skeletal knee kinematics, to identify factors associated with postoperative skeletal kinematic parameters.

**Methods:**

Seventeen knees in 17 patients were prospectively recruited before primary TKA for advanced medial knee osteoarthritis. Subjects underwent BCS-TKA and were evaluated more than 1 year postoperatively. In vivo dynamic skeletal knee kinematics were evaluated using periodic radiographic images collected during squatting to quantify the tibiofemoral functional extension/flexion angle, anteroposterior (AP) translation, and axial rotation angle using image-matching techniques. Rotational alignments of femoral and tibial components were measured postoperatively using computed tomography images.

**Results:**

The pre- and postoperative tibiofemoral functional extension/flexion angles during squatting were 12.2° ± 6.7°/100.1° ± 16.8° and 9.6° ± 8.6°/109.4° ± 16.8°, respectively, with a significant difference in flexion angle (*p* < .05). Total AP translation was significantly larger postoperatively than preoperatively (10.8 mm ± 3.7 mm vs. 14.4 mm ± 4.2 mm, respectively; *p* < .05). The pre- and postoperative total rotation angles were 6.6° ± 3.0° and 6.4° ± 3.7°, respectively, indicating no significant difference. The pre- and postoperative tibiofemoral functional flexion angles were significantly associated with each other (*p* = .0434, *r* = .49). The postoperative total rotation angle was significantly smaller when the total component rotational mismatch angle between the femoral and tibial components was above 5° vs. below 5° (4.6° ± 2.7° vs. 8.3° ± 3.9°, respectively; *p* < .05).

**Conclusions:**

BCS-TKA significantly increased the tibiofemoral functional flexion angles, with larger AP translation postoperatively. Both preoperative skeletal kinematics and surgical techniques affected the skeletal kinematics of the replaced knee. A total component rotational mismatch angle greater than 5° significantly decreased postoperative total knee rotation during squatting.

## Background

Total knee arthroplasty (TKA) is among the most effective therapies to relieve pain and restore knee joint function in patients with advanced knee osteoarthritis (OA). Long-term prosthesis survival rates of TKA have improved due to innovations in surgical techniques and implant design and materials [1]. However, patient satisfaction remains approximately 70–80 % after TKA [2], and is a target for improvement. Previous studies revealed that physiological knee kinematics improved functional performance in patients with TKA [3, 4], while non-physiological knee kinematics [5], specifically decreased or paradoxical tibiofemoral anterior-posterior (AP) translation and axial rotation, worsened clinical outcomes after TKA [4, 6].

Bicruciate-stabilized TKA (BCS-TKA) is designed to achieve more physiological kinematics with asymmetrical femoral condyles and tibial baseplate with concave medial and convex lateral polyethylene articular surfaces, and the dual cam-post mechanism by alternating the function of both the anterior and posterior cruciate ligaments [7]. However, no studies have directly evaluated kinematic changes during squatting before and after BCS-TKA [8, 9]. Even regarding other TKA designs, few *in vivo* studies have assessed the kinematics of replaced or OA knees, or reported how postoperative kinematics are influenced by those preoperatively [6, 10]. Therefore, it is important to determine what factors affect kinematic changes between the pre- and postoperative state in the same knee. Previous studies demonstrated a wide variation of tibiofemoral axial rotation with flexion even after posterior cruciate-substituting TKA under weight-bearing conditions [11–13]. Postoperative rotational kinematics might be significantly affected by preoperative kinematics, as well as by rotational alignment of the tibial component due to the relatively restrictive BCS design.

This prospective study investigated the effects of TKA on skeletal kinematic changes between pre- and postoperative knees. The primary aim of this study was to identify how BCS-TKA affected the total femoral AP translation and axial rotation angle relative to the tibia during squatting in patients with advanced medial knee OA. The secondary aim was to assess the effect of preoperative skeletal kinematics and surgical techniques, specifically the component rotational alignment, on the skeletal kinematics of the replaced knee.

## Methods

### Subjects

The study cohort consisted of 17 knees in 17 patients. All patients were randomly and prospectively recruited before primary TKA for advanced medial knee OA between December 2014 and July 2019 at our institution. The inclusion criteria were as follows: (1) primary TKA; (2) age ≥ 20 years and ≤ 80 years; (3) ability to provide consent and write; and (4) willingness to participate in the study. The exclusion criteria were as follows: (1) rheumatoid arthritis; (2) valgus knee; (3) any history of surgery, fracture, or symptoms in other joints or the spine; (4) severe extra-articular deformity; (5) insufficient bone mineral density (e.g., corticosteroid-induced metabolic bone disease); (6) neuromuscular disease; and (7) systematic or local infection. All patients underwent BCS-TKA (JOURNEY II, Smith & Nephew, Memphis, TN, USA) and were followed up for at least 11 months postoperatively. The study protocol was approved by our Institutional Review Board (numbers 24–166 and 28–375). Written informed consent for participation was submitted by all patients. Patient information is summarized in Table 1 [14].

**Table 1:** Demographic data for all participants

Total knees/participant (n)	17/17
Age (years)	73.1 ± 6.9 (51–79)
Sex (male/female, n)	2/15
BMI (kg/m^2^)	25.6 ± 3.1 (21.9–35.6)
Kellgren-Lawrence Grade [14]	All IV
Follow-up period (months)	17.8 ± 8.8 (11–36)

Values are expressed as the mean ± standard deviation and range

*BMI* body mass index

### Surgical techniques

All TKAs were performed as previously described [15], with a standardized method involving a medial parapatellar approach and modified measured resection technique. Two experienced surgeons performed TKAs using the same technique, and in each operation one individual was the main surgeon while the other was the supervisor, or vice versa. In brief, the femoral and tibial components were aligned perpendicular to their respective mechanical axes in the coronal plane. In the sagittal plane, the tibial components were placed using extramedullary guides to obtain 3° posterior tilting. In the axial plane, the femoral component was aligned parallel to the surgical trans-epicondylar axis (sTEA), while for the tibial AP axis (the line connecting the center of the posterior cruciate ligament at its tibial attachment and the medial border of the patellar tendon at its tibial attachment), the tibial plate was aligned with the femoral component, with verification of rotational mismatch performed using a self-adjusting technique [16, 17]. Soft tissue balancing achieved near-normal medial stability and lateral laxity in knee extension and flexion [18].

### Radiographic measurements

The hip-knee-ankle (HKA) angle was defined as the coronal angle between the mechanical axis of the femur and tibia, using whole-leg radiographs with the patella facing forward (+, varus; -, valgus). The coronal femoral component angle was defined as the medial angle between the mechanical axis of the femur and the horizontal line drawn between the medial and lateral femoral condyles, and was corrected by subtracting the 3° lateral incline of the articular surfaces of the femoral component. The coronal tibial angle was defined as the medial angle between the mechanical axis of the tibia and the horizontal axis of the tibial component. The sagittal femoral angle was defined as the angle between the anatomical axis of the distal femur and a line perpendicular to the distal point of the femoral component. The sagittal tibial angle was defined as the angle between the anatomical axis of the proximal tibia and a horizontal line drawn across the tibial tray [7]. Rotational alignments of femoral and tibial components were measured　postoperatively using computed tomography (CT) (Aquilion, Toshiba, Tochigi, Japan) images obtained at 2-mm intervals between the hip joint and ankle joint [19]. CT images were acquired as DICOM data, and a three-dimensional (3D) image of the lower extremity was reconstructed using 3D template software (version 03.12.03, Kyocera, Kyoto, Japan). Regarding the femoral component, the rotational axis was defined as the sTEA [20]. The rotational alignment of the femoral component was measured as the angle formed by the sTEA and the line joining the anterior margins of the femoral component. The angle was positive if the femoral component was externally rotated compared to the target angle, and negative if it was internally rotated. Regarding the tibial component, the rotational axis was defined as the tibial AP axis [21], which was the line connecting the middle of the posterior cruciate ligament to the medial edge of the patellar tendon, both at their tibial attachments. The tibial AP axis was first identified on a preoperative CT image, then accurately projected onto the postoperative CT image. The rotational alignment of the tibial component was measured as the angle formed by the AP axis of the tibia and the AP axis of the tibial component. The value was positive if the tibial component was externally rotated compared to the target angle, and negative if it was internally rotated. The total component rotational mismatch angle was defined as the sum total of the rotational alignments of the femoral and tibial components, and is represented as an absolute value. The non-weight-bearing femoral rotation angle relative to the tibia was that formed by the AP axis of the tibia and the perpendicular line of the sTEA on pre- or postoperative CT with the patient in a supine position. The value was positive if the femur was externally rotated relative to the tibia, and negative if it was internally rotated. All of these radiographic measurements were repeated three times at least a week apart by one examiner (an orthopaedic surgeon) in all patients, and the average of the three measurements was adopted as the final result. To evaluate intra- and interobserver reproducibility, measurements of all knees were performed three times by one examiner and once by two examiners; all examiners were orthopaedic surgeons. The intra- and interclass correlation coefficients (ICCs) were 0.85/0.86 and 0.87/0.91 for measurement of the coronal femoral and tibial component angles, respectively; 0.95/0.91 and 0.93/0.91 for measurement of the sagittal femoral and tibial component angles, respectively; 0.97/0.93 and 0.97/0.92 for measurement of the pre- and postoperative HKA angles, respectively; 0.93/0.87 and 0.86/0.82 for measurement of the pre- and postoperative non-weight-bearing femoral rotation angles relative to the tibia, respectively; and 0.83/0.83 and 0.86/0.82 for measurement of the femoral and tibial component rotational angles, respectively. ICC values between 0.75 and 0.90 indicate good reliability, while values greater than 0.90 represent excellent reliability [22].

### Clinical measurements

Patient-reported outcomes using the KSS 2011 [23] were assessed before and at least 1 year after surgery. The KSS 2011 consists of four subscales: symptoms, satisfaction, expectations, and functional activities. Functional activities are evaluated by four subcategories: walking and standing, standard activities, advanced activities, and discretionary activities. The maximum score of each subscale is 25 for symptoms, 40 for satisfaction, and 100 for functional activities.

### Kinematic evaluation

The procedures were performed using a previously validated, computer-assisted image-matching procedure [7, 24]. Continuous radiographic images at a frame rate of 10 Hz were obtained during squatting using a flat-panel X-ray detector (FPD; Ultimax-I, Toshiba, Tochigi, Japan), with an image area of 420 mm (H) × 420 mm (V), resolution of 0.274 mm × 0.274 mm/pixel, 0.02 s pulse width, 80 kV, and 360 mA.

**Fig. 1:**
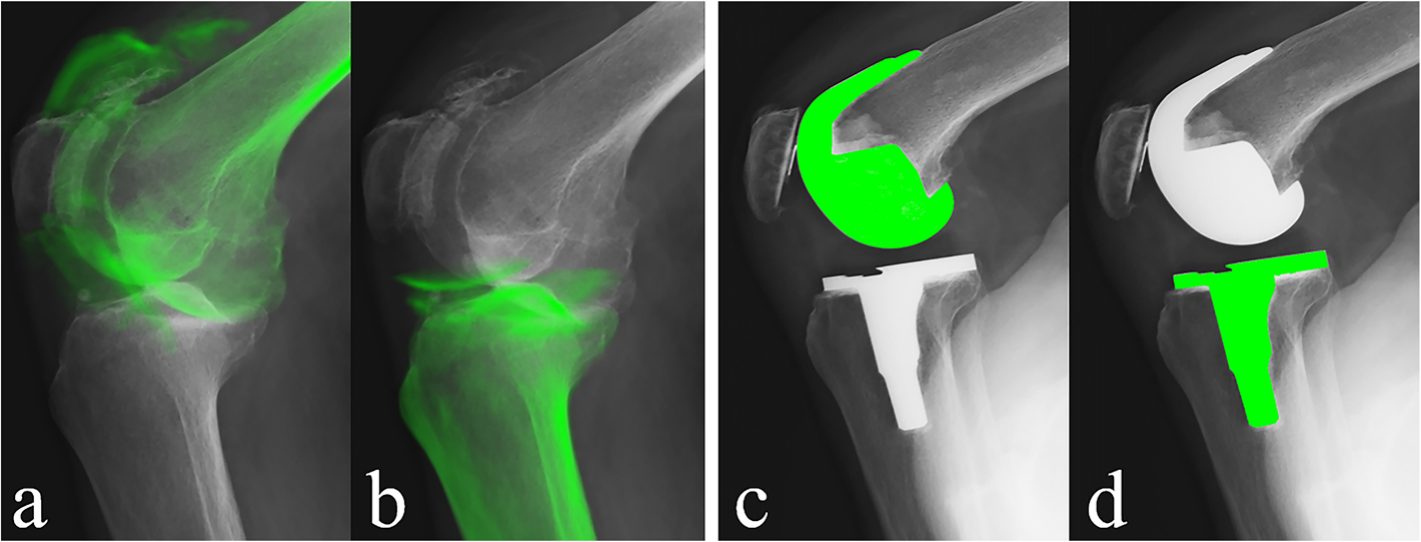
Computed tomography slices were used to create density-based digitally reconstructed radiographs (DDR) of the femur (**a**) and the tibia (**b**), which were projected onto the radiographic images of the preoperative knee. Computer-aided design data of the femoral (**c**) and tibial (**d**) components were also projected onto radiographic images of postoperative knee

**Fig. 2:**
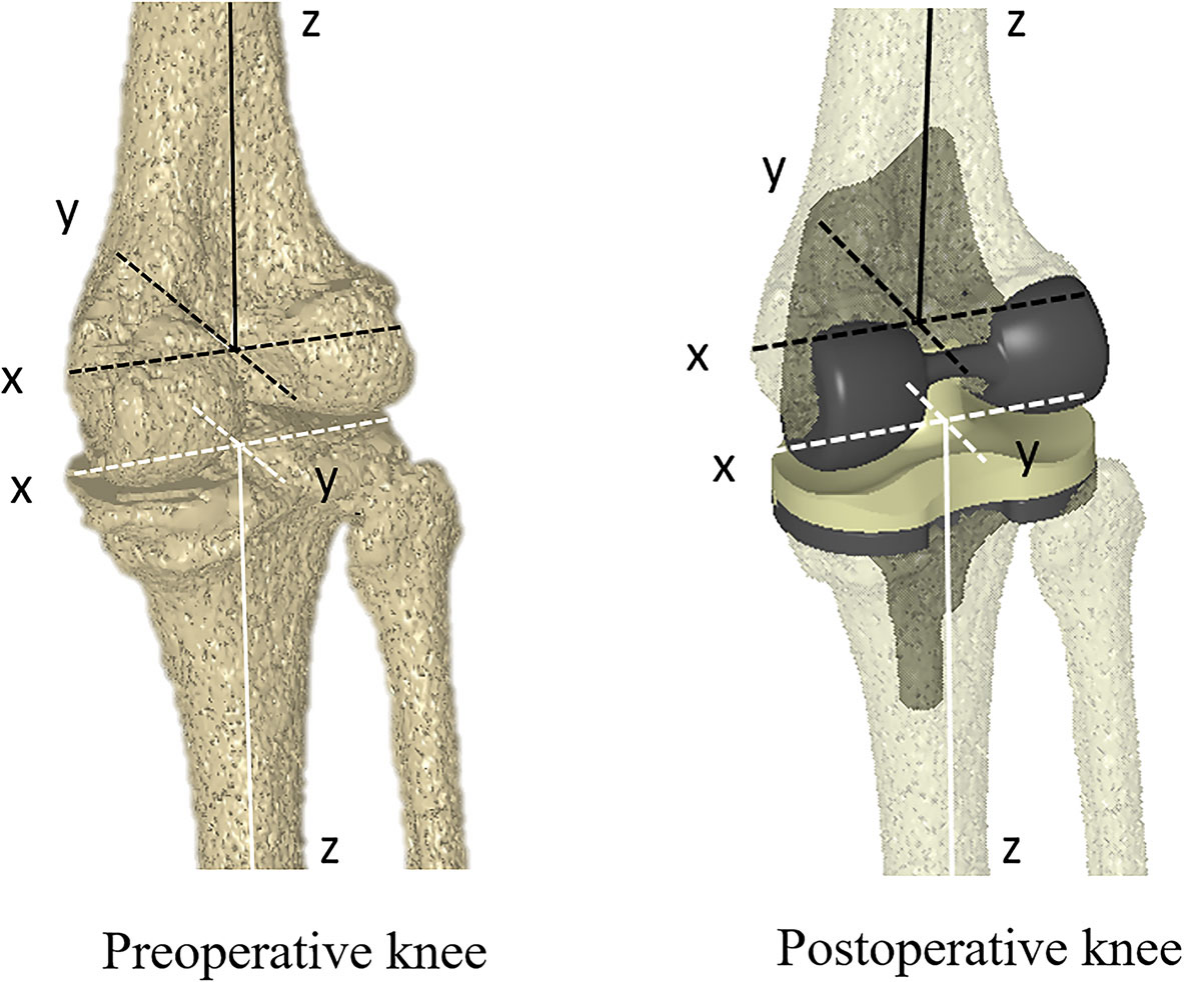
Density-based volumetric bone models of preoperative, postoperative knees showing identical embedded anatomic coordinate systems

For the preoperative knee, a 3D greyscale digital bone model was generated from CT images with a 512 × 512 image matrix, 0.35 mm × 0.35 mm/pixel, and 1-mm thickness, and density-based image-matching techniques were used to evaluate the 3D positions and orientations of the femur and tibia in each radiographic image obtained with the FPD (Fig. 1) [25, 26]. Anatomic coordinate systems were embedded in each density-based volumetric bone model (Fig. 2). The coordinate system of the femur was defined as follows: the midpoint of the sTEA was the origin, the sTEA was the mediolateral (x) axis, the distal anatomical axis of the femur was the proximal/distal (z) axis, and the line perpendicular to the x and z axes was the AP (y) axis. The coordinate system of the tibia was defined as follows: the intercondylar eminence of the tibia was the origin, the line parallel to the proximal anatomical axis of the tibia was the proximal/distal (z) axis, the line connecting the middle of the posterior cruciate ligament to the tibial attachment of the medial edge of the patellar tendon was the AP (y) axis, and the line perpendicular to both axes was the mediolateral (x) axis. Regarding the postoperative knee, image-matching techniques were performed using the 3D greyscale digital bone model generated from the CT images and the manufacturer-provided 3D computer-aided design models of the femoral and tibial components. First, the 3D positions and orientations of the femoral and tibial components were evaluated as described previously [27] (Fig. 1), and the 3D greyscale digital bone models were concurrently projected and superimposed onto each two-dimensional (2D) radiographic image. Next, the 3D positions and orientations of the femoral and tibial components were converted to the previously described skeletal anatomical coordinate system of the femur and tibia to enable comparison with the preoperative skeletal kinematics obtained using the same anatomical coordinate system.

We evaluated the following *in vivo* pre- and postoperative 3D skeletal knee kinematic parameters of each subject: tibiofemoral functional extension/flexion angle (flexion +, extension –), femoral AP position (anterior +, posterior –), and axial rotation angle (external +, internal –) relative to the tibia. The total femoral AP translation and rotation angle relative to the tibia were respectively calculated as the AP translation and axial rotational movement of the femur relative to the tibia during squatting. The maximal posterior position of the femur relative to the tibia was defined as the most posterior position of the femur during full squats. Previous studies [24, 25, 28] estimated the root-mean-square (RMS) accuracy errors for this method as follows: for the femur/tibia in non-replaced knees, 0.12 mm/0.15 mm for in-plane translation, 0.11 mm/0.10 mm for out-of-plane translation, and 0.27°/0.30° for rotation [24, 25]; for the femoral/tibial component in replaced knees, 0.11 mm/0.13 mm for in-plane translation, 0.26 mm/0.18 mm for out-of-plane translation, and 0.19°/0.22° for rotation [28].

### Statistical analysis

All statistical analyses were performed using JMP software (Version 14.0, SAS Institute Inc., Cary, NC, USA), except for sample size analysis, which was conducted using EZR (Saitama Medical Center, Jichi Medical University, Saitama, Japan), a graphical user interface for R (The R Foundation for Statistical Computing, Vienna, Austria) [29]. The Wilcoxon signed rank test was used to evaluate differences in KSS 2011 scores between pre- and postoperative knees. The paired t-test was used to analyze differences between pre- and postoperative knees in terms of the HKA angle, non-weight-bearing femoral rotation angle relative to the tibia, tibiofemoral functional extension/flexion angle, total femoral AP translation relative to the tibia, femoral AP position relative to the tibia at each knee flexion angle, total femoral rotation angle relative to tibia, and femoral rotation angle relative to the tibia at each knee flexion angle. The paired t-test was also used to evaluate the difference in the postoperative non-weight-bearing femoral rotation angle relative to the tibia (CT images) and the postoperative tibiofemoral rotation angle during the first standing position before squatting (image-matching technique). Spearman’s correlation analysis was used to evaluate correlations between pre- and postoperative tibiofemoral functional extension/flexion angles and the total AP translation and rotation angle relative to the tibia. Spearman’s correlation analysis was conducted to evaluate the correlation between the preoperative non-weight-bearing femoral rotation angle relative to the tibia and the total femoral rotation angle relative to the tibia. All Spearman’s correlation analyses were performed using a 95 % confidence ellipse. The non-paired t-test was used to compare the postoperative tibiofemoral functional extension/flexion angle and the postoperative total femoral AP translation and rotation angle relative to the tibia between normal (≤ 5°) and malalignment (> 5°) groups defined in terms of the total component rotational mismatch angle. In all statistical analyses, significance was defined by *p* < .05. The primary outcomes of this study were the total femoral AP translation and rotation angle relative to the tibia. A sample size calculation showed that 16 knees per group would permit detection of a 3.5-mm difference in the total femoral AP translation relative to the tibia or a 3.5° difference in the total femoral rotation angle relative to the tibia (power = 0.8, α = 0.05) between the pre- and postoperative states, with standard deviations of 4.5 mm and 4.5°, respectively.

## Results

### Radiographic and clinical outcomes

Pre- and postoperative radiographic and clinical information are presented in Table 2. The HKA angle and the score for three KSS 2011 subscales, symptoms, satisfaction, and functional activities, were significantly improved postoperatively.

**Table 2:** Clinical and radiographic data for pre- and postoperative knees

	preoperative	postoperative	*p*-value
HKA angle (°)	11.9 ± 4.4 (3 to 23)	1.5 ± 1.9* (-4 to 5)	< 0.001
Coronal femoral component angle (°)	N/A	91.5 ± 2.0 (88 to 96)	
Coronal tibial component angle (°)	N/A	89.7 ± 1.7 (86 to 93)	
Sagittal femoral component angle (°)	N/A	89.7 ± 2.4 (85 to 94)	
Sagittal tibial component angle (°)	N/A	85.0 ± 2.1 (81 to 90)	
Total component rotational mismatch angle (°)	N/A	5.4 ± 4 (0.7 to 15.1)	
Axial femoral component angle (°)	N/A	-0.6 ± 3.1 (-4.7 to 5.3)	
Axial tibial component angle (°)	N/A	0.6 ± 4.7 (-5.5 to 10.4)	
KSS 2011 [18]			
Symptoms (25)	5.5 ± 3.4 (0 to 12)	22.8 ± 2.6* (17 to 25)	< 0.001
Satisfaction (40)	12.6 ± 5.1 (4 to 22)	30.4 ± 7.8* (16 to 40)	< 0.001
Expectations (15)	13.6 ± 1.5 (10 to 15)	10.7 ± 2.9* (6 to 15)	0.003
Functional activities (100)	38.1 ± 16 (18 to 69)	75.1 ± 14* (47 to 94)	< 0.001

Values are expressed as the mean ± standard deviation and range

*HKA* hip-knee-ankle, *KSS* knee society score, *N/A* not applicable

*significantly different between pre- and postoperative knee (*p* < .05)

The mean total component rotational mismatch angle was 5.4° ± 4° in all 17 knees, and in nine knees this angle was larger than 5°, indicating malalignment (normal vs. malalignment groups: 2° ± 1° vs. 8.5° ± 3.1°, respectively, *p* < .0001) (Table 2). The mean pre- and postoperative non-weight-bearing femoral rotation angles relative to the tibia were significantly different, as shown in Table 3.

**Table 3:** Kinematic and CT data for pre- and postoperative knees

	preoperative	postoperative	*p*-value
Tibiofemoral functional extension angle (°)	12.2 ± 6.7	9.6 ± 8.6	0.3389
Tibiofemoral functional flexion angle (°)	100.1 ± 16.8	109.4 ± 16.8*	0.0037
Total femoral AP translation relative to the tibia (mm)	10.8 ± 3.7	14.4 ± 4.2*	0.0093
Maximal posterior position of the femur relative to the tibia (mm)	-7.4 ± 4.7	-13.1 ± 4.9*	< 0.001
Total femoral rotation angle relative to the tibia (°)	6.6 ± 3.0	6.4 ± 3.7	0.8495
Non-weight-bearing femoral rotation angle relative to the tibia (°)	-0.6 ± 4.8	7 ± 4.5*	< 0.001

Values are expressed as the mean ± standard deviation and range

*AP* anterior-posterior

*significantly different between pre- and postoperative knees (*p* < .05)

### Primary aim: kinematic outcomes

**Fig. 3:**
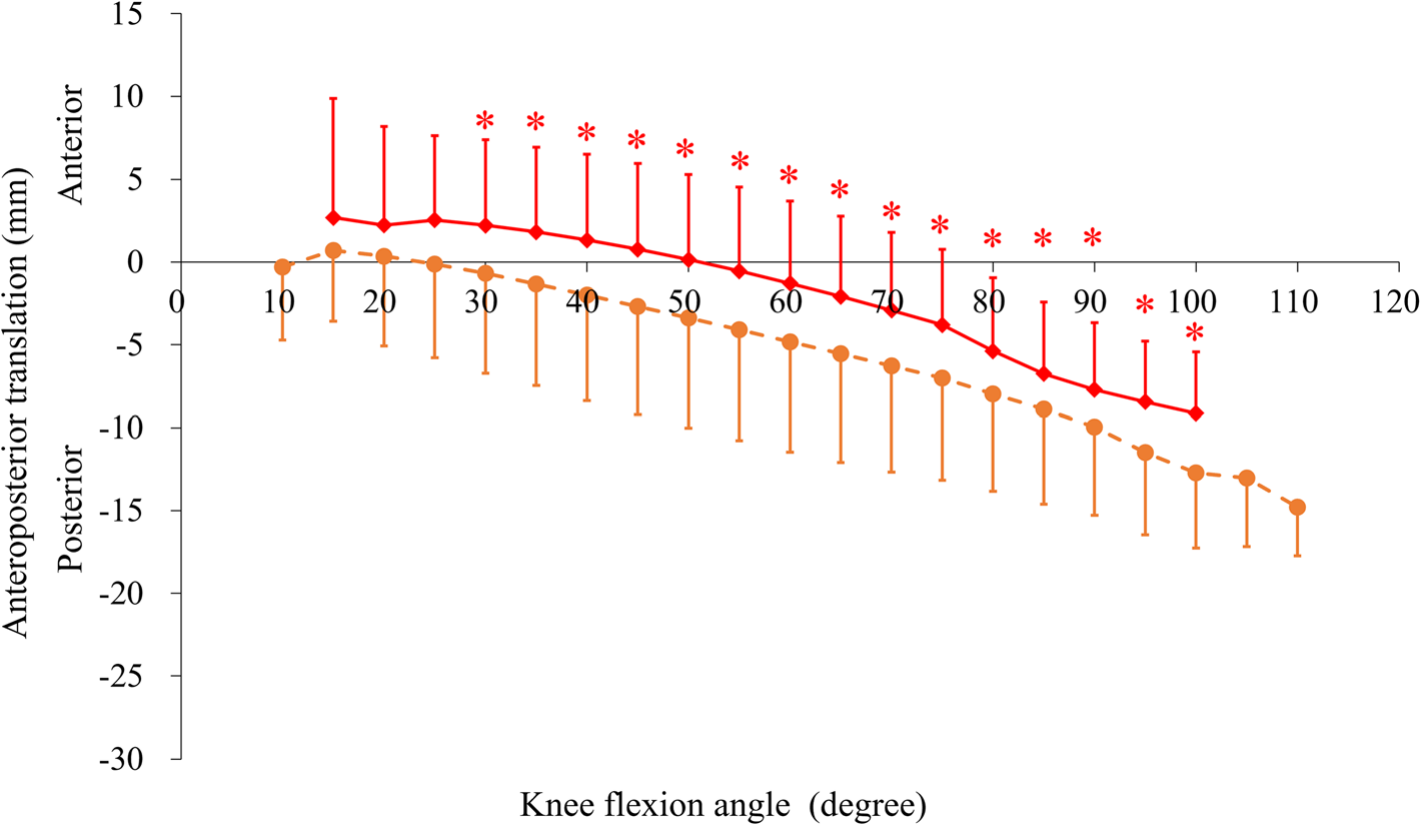
Mean anteroposterior translation of the femur relative to the tibia during squatting. The solid-red and dashed-orange lines indicate preoperative and postoperative knees, respectively. *p < .05

**Fig. 4:**
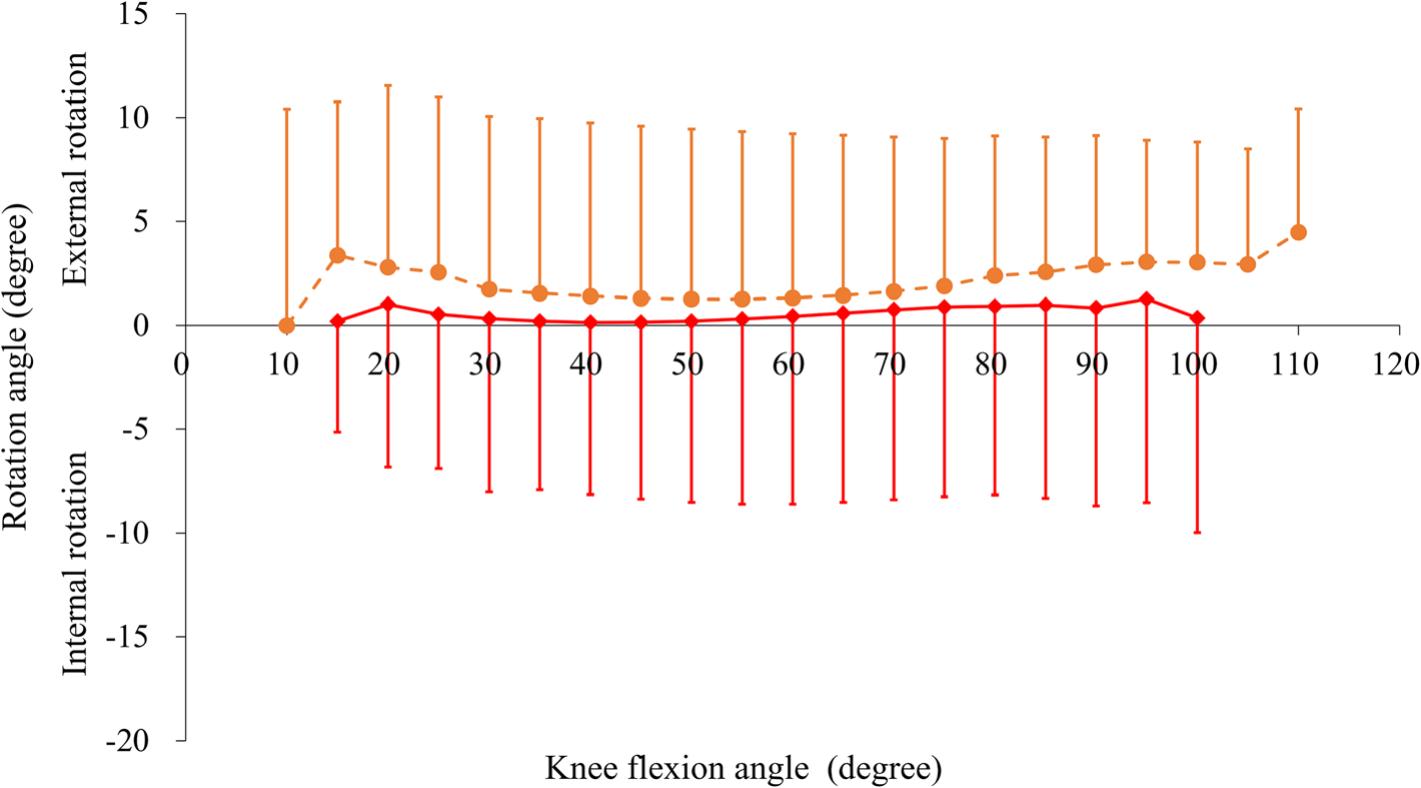
Mean rotation angle of the femur relative to the tibia during squatting. The solid-red and dashed-orange lines indicate preoperative and postoperative knees, respectively

**Fig. 5:**
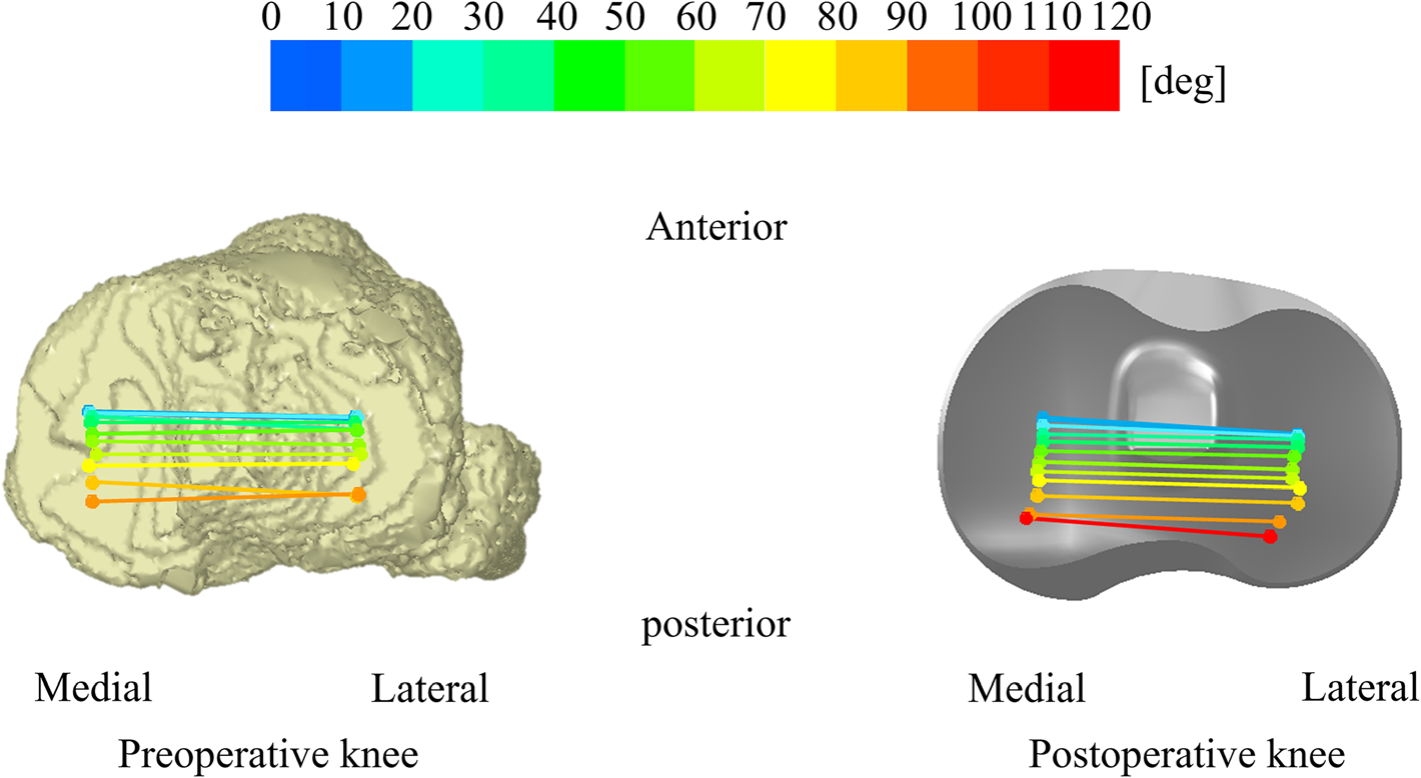
Mean movement of the femoral surgical trans-epicondylar axis of preoperative and postoperative knees projected onto the axial plane of the tibia during squatting

Skeletal knee kinematics (tibiofemoral functional extension/flexion angle, femoral AP position, and axial rotation angle relative to the tibia) are shown in Table 3; Figs. 3, 4 and 5.

The tibiofemoral functional flexion angles, but not the functional extension angles, differed significantly between the pre- and postoperative states (Table 3).

The total femoral AP translation relative to the tibia changed significantly increased (Table 3). The femoral AP position relative to the tibia became increasingly more posterior postoperatively than preoperatively for every 5° interval of tibiofemoral functional flexion from 30° to 100° (*p* < .05 for each; Fig. 3). The maximal posterior position of the femur relative to the tibia was significantly more posterior postoperatively than preoperatively (Table 3).

The pre- and postoperative total femoral rotation angles relative to the tibia showed no significant difference (Table 3; Fig. 4). There was also no significant difference between the pre- and postoperative femoral rotation angles at each tibiofemoral functional flexion angle (Fig. 4).

### Secondary aim: pre- and postoperative kinematics and component rotational alignment

The pre- and postoperative tibiofemoral functional flexion angles were significantly associated with each other (*p* = .0434, *r* = .49). The postoperative tibiofemoral functional extension angle and total femoral AP translation relative to the tibia were not significantly associated with the respective preoperative values (*p* > .05 for each). The pre- and postoperative total femoral rotation angles relative to the tibia were not significantly correlated (*p* = .08), but there was a weak positive trend (*r* = .44).

The postoperative tibiofemoral functional flexion angle was significantly associated with the postoperative total femoral AP translation relative to the tibia (*p* = .0084, *r* = .62) and with the postoperative maximal posterior position of the femur relative to the tibia (*p* = .0236, *r* = -.55).

The preoperative non-weight-bearing femoral rotation angle relative to the tibia was not significantly correlated with the preoperative total femoral rotational angle relative to the tibia (*p* = .2395). The postoperative non-weight-bearing femoral rotation angle relative to the tibia (7.2° ± 4.1°) was significantly different from the postoperative tibiofemoral rotation angle during the first standing position before squatting (2.1° ± 9.1°; *p* = .0426). In terms of component rotational alignment, the normal group demonstrated a significantly greater total femoral rotation angle relative to the tibia (8.3° ± 3.9°) than the malalignment group (4.6° ± 2.7°; *p* = .0408).

## Discussion

This study investigated the *in vivo* skeletal kinematic differences during squatting between pre- and postoperative knees after TKA. BCS-TKA significantly changed the tibiofemoral functional flexion angle, AP position, and total AP translation relative to the tibia compared to the preoperative state. Both preoperative skeletal kinematics and surgical techniques affected postoperative skeletal kinematics during squatting. Pre- and postoperative tibiofemoral functional flexion angles were significantly correlated, and postoperative kinematic patterns were similar to those in pathological OA knees. Regarding surgical technique, component rotational malalignment was associated with a significantly reduced postoperative total femoral rotation angle relative to the tibia.

Deficiency of the anterior cruciate ligament can cause paradoxical anterior translation of the femur relative to the tibia [9]. In a previous study, cruciate-retaining and posterior-stabilized TKAs showed less total AP translation relative to the tibia (approximately 6–12 mm) than BCS-TKA in the present study [30, 31]. This study, which was the first to directly compare pre- and postoperative skeletal kinematics in individual patients, also showed that knees replaced using BCS-TKA demonstrated a significantly posterior femoral position relative to the tibia, with greater total AP translation relative to the tibia (14.4 mm) than preoperative OA knees (10.8 mm). Consistent with the current findings, greater posterior femoral rollback was associated with increased knee flexion because it delayed impingement between the femur and the posterior part of the tibial component [32]. Regarding the tibiofemoral functional flexion angle, a previous study reported that BCS-TKA demonstrated a 5° increase relative to preoperative OA knees, suggesting improvement and supporting the results of our study [33]. However, the postoperative total AP translation and tibiofemoral functional flexion angle in this study remained significantly smaller than those of normal knees. The healthy knee exhibits 23.2 mm of posterior femoral rollback during squatting from 0° to 140° of knee flexion [25]. Ligament stiffness and soft tissue contractures in knees with advanced OA may still reduce postoperative total femoral AP translation relative to the tibia during dynamic weight-bearing knee flexion [34].

In this study, skeletal axial kinematics measured using the sTEA showed that BCS-TKA could not restore skeletal knee kinematics to their physiological state. The postoperative total femoral rotation angle (6.4°) during squatting remained lower than that in healthy knees (16.1°) [25]. Furthermore, the postoperative femoral rotational angles and kinematic patterns for every 5° interval of knee flexion were similar to those preoperatively. In previous studies, OA knees showed significantly decreased rotation during activities involving knee flexion [25, 34] or twisting [26], which is consistent with the preoperative total femoral rotation (6.6°) in the current study. In a study based on CT images, Kawaguchi et al. reported that inappropriate preoperative tibiofemoral rotational alignment and rotational alignment of the tibial and femoral components were risk factors for postoperative component rotational mismatch under non-weight-bearing conditions [35]. The total component rotational mismatch angle can also affect postoperative skeletal knee kinematics. In this study, when this angle was over 5° it significantly reduced the postoperative total femoral rotation angle by an average of 3.7°. Previous studies evaluated the effect of this angle on rotational kinematics [11, 12, 36]. Harman et al. reported that relative femoral-tibial component rotational mismatch of over 5° caused a significantly smaller rotational angle during knee flexion [11], consistent with the present study. Nakahara et al. also reported that the fixed-bearing surface of the implant did not fully compensate for the rotational malalignment of the tibial component during weight-bearing conditions [12]. Lützner et al. reported that a mismatch over 10° significantly reduced femoral rotation during knee flexion and worsened functional scores [36]. Surgeons should avoid rotational mismatch because it affects postoperative skeletal kinematics and possibly also clinical outcomes [36].

This study has several limitations. First, knee kinematics such as AP translation and axial rotation could vary based on the activity performed [24–26]. However, squatting is a frequent and important activity in daily life even after TKA. Second, we did not evaluate the correlations between the postoperative kinematics and the postoperative KSS 2011 scores. The study may have been underpowered (Type II error) to detect such correlations, because the study was powered to detect an estimated significant difference in the primary outcome, namely the total femoral AP translation and rotation angle relative to the tibia. However, there were significant postoperative improvements in the tibiofemoral functional flexion angle during squatting, as well as in the score for three KSS 2011 subscales: symptoms, satisfaction, and functional activities. Future studies should determine how skeletal kinematics affect objective measurements such as knee muscle strength and balance function, because many factors influence patient-reported clinical outcome scores [5]. Third, several factors may influence the postoperative femoral rotation angle relative to the tibia, including variations in the preoperative rotational relationship, component alignment [12, 35], soft tissue balance, conformity [12], presence or absence of load, and posture. Further investigations with sufficient sample sizes are necessary to examine the potential impact of these factors.

## Conclusions

To the best of our knowledge, this study is the first to directly evaluate changes in *in vivo* skeletal kinematics before and after BCS-TKA. Relative to the preoperative state, BCS-TKA showed significantly more posterior femoral AP position and greater total femoral AP translation with an increased tibiofemoral functional flexion angle during squatting. Both preoperative skeletal kinematics and surgical techniques affected the skeletal kinematics of the replaced knee under weight-bearing conditions. The pathological skeletal kinematics of OA knees in flexion and rotation influenced the postoperative skeletal kinematics of flexion and rotation, respectively. A total component rotational mismatch angle over 5° significantly reduced the postoperative total femoral rotation relative to the tibia during squatting. These findings provide insight into how postoperative skeletal knee kinematics are affected by preoperative skeletal knee kinematics, rotational alignment, and implant design.

## Data Availability

The datasets supporting the conclusions of the present study are available from the corresponding author on reasonable request.

## Abbreviations

BCS-TKA: Bicruciate-stabilized total knee arthroplasty
AP: Anteroposterior/Anterior-posterior
TKA: Total knee arthroplasty
OA: Osteoarthritis
BCS: Bicruciate-stabilized
BMI: Body mass index
sTEA: Surgical trans-epicondylar axis
HKA: Hip-knee-ankle
CT: Computed tomography
3D: Three-dimensional
ICCs: Intra- and interclass correlation coefficients
KSS 2011: Knee Society Score 2011
FPD: Flat-panel X-ray detector
DDR: Density-based digitally reconstructed radiographs
2D: Two-dimensional
RMS: Root-mean-square
N/A: Not applicable
